# NIR-II live imaging study on the degradation pattern of collagen in the mouse model

**DOI:** 10.1093/rb/rbac102

**Published:** 2022-12-13

**Authors:** Huizhu Li, Xinxian Meng, Huaixuan Sheng, Sijia Feng, Yuzhou Chen, Dandan Sheng, Liman Sai, Yueming Wang, Mo Chen, Yan Wo, Shaoqing Feng, Hossein Baharvand, Yanglai Gao, Yunxia Li, Jun Chen

**Affiliations:** Department of Sports Medicine, Sports Medicine Institute of Fudan University, Huashan Hospital, Fudan University, Shanghai 200040, China; Department of Plastic and Reconstructive Surgery, School of Medicine, Shanghai Jiao Tong University, Shanghai Ninth People’s Hospital, Shanghai 200011, China; Department of Sports Medicine, Sports Medicine Institute of Fudan University, Huashan Hospital, Fudan University, Shanghai 200040, China; Department of Sports Medicine, Sports Medicine Institute of Fudan University, Huashan Hospital, Fudan University, Shanghai 200040, China; Department of Sports Medicine, Sports Medicine Institute of Fudan University, Huashan Hospital, Fudan University, Shanghai 200040, China; Department of Sports Medicine, Sports Medicine Institute of Fudan University, Huashan Hospital, Fudan University, Shanghai 200040, China; Department of Physics, Shanghai Normal University, Shanghai 200234, China; Department of Anatomy and Physiology, School of Medicine, Shanghai Jiao Tong University, Shanghai 200025, China; Department of Sports Medicine, Sports Medicine Institute of Fudan University, Huashan Hospital, Fudan University, Shanghai 200040, China; Department of Anatomy and Physiology, School of Medicine, Shanghai Jiao Tong University, Shanghai 200025, China; Department of Plastic and Reconstructive Surgery, School of Medicine, Shanghai Jiao Tong University, Shanghai Ninth People’s Hospital, Shanghai 200011, China; Department of Stem Cells and Developmental Biology, Cell Science Research Center, Royan Institute for Stem Cell Biology and Technology, ACECR, Tehran 1665659911, Iran; Department of Developmental Biology, School of Basic Sciences and Advanced Technologies in Biology, University of Science and Culture, Tehran 1461968151, Iran; Hexi College, Zhangye, Gansu 73400, China; Department of Sports Medicine, Sports Medicine Institute of Fudan University, Huashan Hospital, Fudan University, Shanghai 200040, China; Department of Sports Medicine, Sports Medicine Institute of Fudan University, Huashan Hospital, Fudan University, Shanghai 200040, China

**Keywords:** collagen, degradation rate, NIR-II live imaging, *in vivo*, crosslinking degree

## Abstract

The degradation of collagen in different body parts is a critical point for designing collagen-based biomedical products. Here, three kinds of collagens labeled by second near-infrared (NIR-II) quantum dots (QDs), including collagen with low crosslinking degree (LC), middle crosslinking degree (MC) and high crosslinking degree (HC), were injected into the subcutaneous tissue, muscle and joints of the mouse model, respectively, in order to investigate the *in vivo* degradation pattern of collagen by NIR-II live imaging. The results of NIR-II imaging indicated that all tested collagens could be fully degraded after 35 days in the subcutaneous tissue, muscle and joints of the mouse model. However, the average degradation rate of subcutaneous tissue (*k* = 0.13) and muscle (*k* = 0.23) was slower than that of the joints (shoulder: *k* = 0.42, knee: *k* = 0.55). Specifically, the degradation rate of HC (*k* = 0.13) was slower than LC (*k* = 0.30) in muscle, while HC showed the fastest degradation rate in the shoulder and knee joints. In summary, NIR-II imaging could precisely identify the *in vivo* degradation rate of collagen. Moreover, the degradation rate of collagen was more closely related to the implanted body parts rather than the crosslinking degree of collagen, which was slower in the subcutaneous tissue and muscle compared to the joints in the mouse model.

## Graphical abstract

**Figure:**
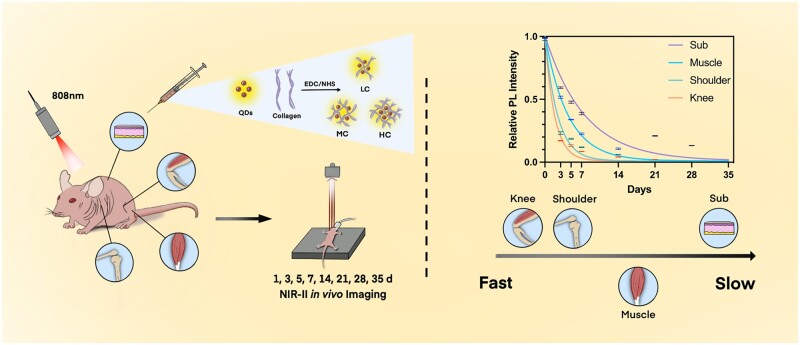

## Introduction

Collagen, as a friendly biomaterial for human body, has been widely employed for biomedical applications, such as disc repair, bone defect, tendon damage and drug delivery [1–7], owing to its suitable biological properties and low immunogenicity [3, 8–10]. As an additional implant, an appropriate degradation rate plays a critical role in its clinical performance. Generally, the ideal degradation of a collagen implant is expected to keep the same pace as the regeneration of the host tissue [11–13]. Rapid degradation would not support the regeneration of the injured tissue sufficiently, which would result in re-tear or re-injury [14, 15]. In contrast, the prolonged degradation period of implants could hinder the host tissue reproduction and increase the infection risk of implants and the surrounding tissue [15]. Therefore, the degradation of collagen is an essential factor for the therapeutic results of collagen-based products in the body.

In fact, the collagen degradation mainly depends on the chemical properties of itself and the biological microenvironment of the implantation sites. Firstly, crosslinking degree is one of the most critical properties that would affect the degradation [16] and the function of the collagen-based products [17, 18]. Previously, the effects of crosslinking degree of collagen on its degradation were mainly studied by *ex vivo* investigation of collagen mass under the exogenous collagenase [8, 17, 19]. Secondly, a key aspect of the microenvironment in the implantation sites is the level of collagenase, which is primarily secreted from macrophages and fibroblasts [20]. A considerable amount of research has investigated *in vivo* collagen degradation in a single body part such as subcutaneous tissue and knee joint [21–27]. However, since the level of cells and secreted collagenase varies in different body parts, investigation in a single body part is somewhat insufficient, neglecting the varying microenvironment. To date, there is little work that simultaneously takes both the crosslinking degree and the different body parts into account for the *in vivo* investigation of collagen degradation.

**Scheme 1.**
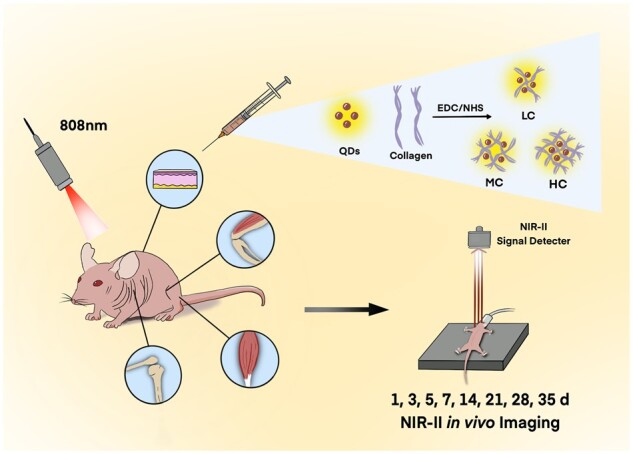
Schematic illustration of monitoring the collagen degradation *in vivo*.

Our study was designed to investigate the role of both the crosslinking degree and the body parts on the collagen degradation (Scheme 1). Notably, second near-infrared (NIR-II) *in vivo* imaging was utilized as a live animal imaging technique to precisely record the dynamic changes of collagen. Firstly, highly fluorescent NIR-II PbS quantum dots (QDs) were conjugated with low crosslinking degree of collagen (LC), middle crosslinking degree of collagen (MC) and high crosslinking degree of collagen (HC), respectively. Three crosslinking degrees of collagens were injected into the subcutaneous tissue, muscle and joints of the mouse model. Then, the NIR-II fluorescence (FL) intensities of LC, MC and HC in the mouse model were monitored and analyzed by NIR-II live animal imaging from 1 h to 35 days post-injection. *Ex vivo* imaging and histological analysis were also employed in order to further assess the degradation properties of collagen.

## Methods

### Reagents and instrumentation

Lead (II) acetate trihydrate [Pb(CH_3_CO_2_)_2_·3H_2_O, ≥99%], sodium sulfide and anhydrous (Na_2_S, ≥90%), were purchased from J&K Scientific (China). Ribonuclease-A from bovine pancreas (>70 U/mg), was purchased from Sigma-Aldrich (USA). Type I collagen from cow calcaneal tendon and sodium hydroxide (NaOH, ≥95%) were bought from Shanghai Macklin Biochemical Co., Ltd. Deionized water was utilized as the working solution. The procedure of microwave synthesis was done with microwave reactor Discover SP (CEM, USA). The NIR-II FL images were obtained from the Artemis Intelligent Imaging system (Shanghai Hengguang Zhiying Medical Technology Co., Ltd.) with 808 nm of excitation source provided by two fiber-coupled lasers. NIR long-pass filter of 1250 nm was used and emitted FL signal was caught by InGaAs camera NIRvana 640 (Princeton, USA).

### Preparation of QDs-labeled collagen

Highly fluorescent protein-encapsulated lead sulfide QDs were synthesized according to our previous method [28]. Briefly, 500 µl of 10 mM CH_3_COOHPb and 500 µl of 50 mg/ml RNase-A were mixed and 1 M NaOH was added to adjust the pH to 9–10. Then 50 µl of 10 mM Na_2_S was added. The tube was put into the microwave reactor immediately at 70°C for 30 s, and the dark brown product was found. Collagen was dissolved in 0.1 M MES (2-(N-morpholino)ethanesulfonic acid) buffer and mixed with different amounts of crosslinkers at room temperature for 2 h according to the previous study [19] and further conjugated with QDs for another 2 h. Specifically, collagen, EDC (1-(3-Dimethylaminopropyl)-3-ethylcarbodiimide hydrchloride) and NHS (N-Hydroxysuccinimide) (1:1.15:2.76) were utilized in order to prepare HC and set as 100% concentration. Besides, 10% and 50% concentrations were utilized for the same process to form LC and MC, respectively. All freshly prepared QDs-labeled collagen was ultra-centrifugated with 30 kDa ultra-centrifugal filter tube to remove excessive reagents and stored at 4°C. Then, PL (Photoluminescence) spectrum and UV (Ultraviolet) spectrum of QDs-labeled collagen were measured.

### *In vivo* NIR-II live animal imaging

Eight-week-old female BALB/C nude mice with the weight of 23–25 g were provided by Shanghai Jiesijie Laboratory Animal Co., Ltd. Related animal studies were carried out in agreement with the guidelines approved by the Animal Care Committee of the Laboratory Animal from Fudan University. Mice were anesthetized using isoflurane at a flow rate of 1.5 l/min, while oxygen at a flow rate of 0.2 l/min was administered simultaneously, throughout injections and *in vivo* imaging. Ten microliters of QDs-labeled collagen (LC, MC and HC) and pure QDs were injected to the back (subcutaneous tissue), shoulder joint cavity, knee joint cavity and hind leg muscle of the nude mice. Mutual interference was avoided between the injection sites. Then NIR-II images were obtained at 1 h, 1, 3, 5, 7, 14, 21, 28 and 35 days after injection as appropriate (28 days for joints). The exposure time was 50 ms for subcutaneous tissue and 100 ms for muscle, the shoulder and knee joint. The FL intensity of injection points from each image was measured using Image J and normalized FL intensity was calculated. As collagen degradation is a kind of *in vivo* metabolism, the exponential decay model (*y* = span**·***e*^−^^*kx*^ + plateau) was utilized for curve fitting by Graphpad Prism 9.3.1 [29, 30], and *k*, as the degradation rate, was calculated as follows in this work:

$$k=\frac{{\ln(\mathrm{relative}\;\mathrm{PL}\;\mathrm{intensity}}^{-1})}{\mathrm{days}}.$$

### Histological analysis and *ex vivo* NIR-II imaging

Skin, muscle, shoulder and knee joints in different groups were harvested to perform histological analysis. Injection points were marked previously under the guidance of the NIR-II imaging system. The whole layer of skin with a size ∼0.5 × 0.2 cm was collected from subcutaneously injected points. As for shoulder joints, the humerus and scapula as well as muscles and ligaments were kept to ensure that intact shoulder joint capsule was taken. Knee joints were harvested in the same way by keeping the femur and tibia as well as surrounding muscles and ligaments. Muscles with a size ∼0.5 × 0.5 cm were taken from the injection point. Masson’s trichrome (Masson) staining was performed for the specimens mentioned above. The major organs including cerebrum, lung, heart, stomach, kidney, spleen, liver and intestine were harvested and NIR-II images were taken 35 days after injection. Histological analysis of major organs from QDs-injected mice and LC-, MC- and HC-injected mice were performed and hematoxylin–eosin (HE) staining was applied.

## Results

### Characterization of NIR-II QDs-labeled collagen

**Figure 1.**
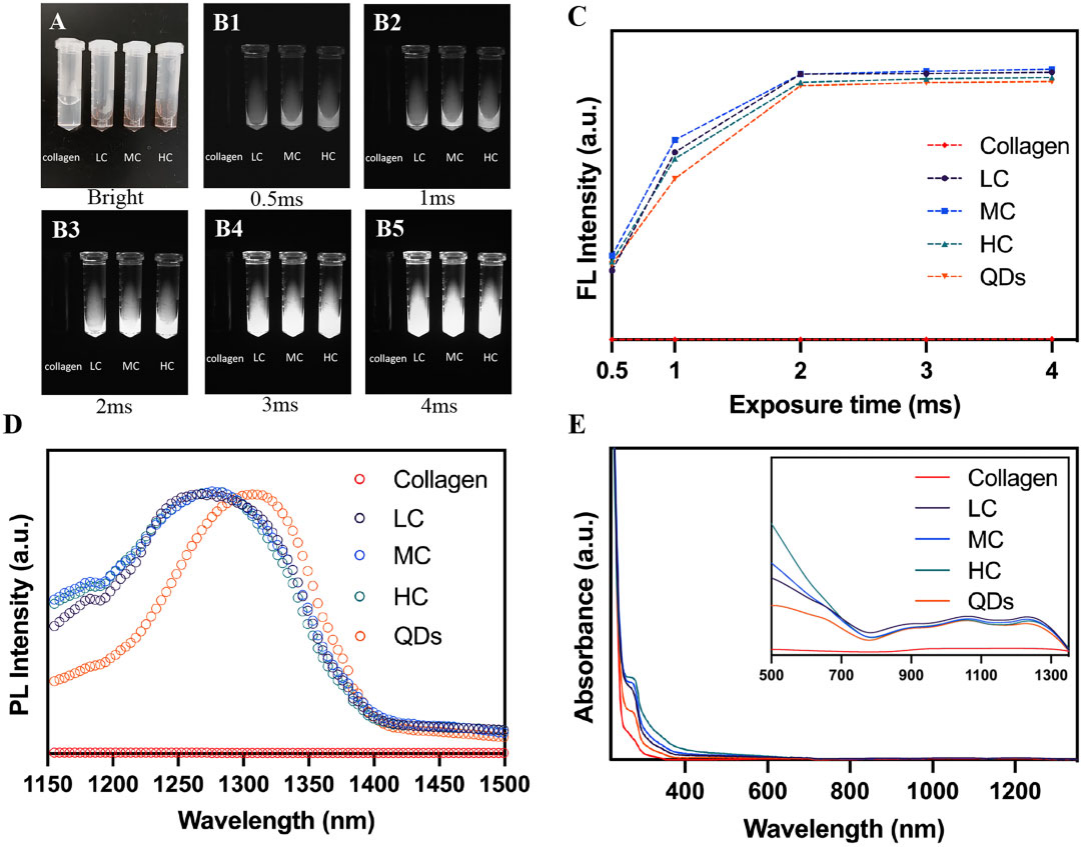
Characterization of QDs-labeled collagen. (**A**) Bright-field and (**B1-B5**) NIR-II images of collagen, LC, MC and HC at different exposure times. Corresponding FL intensity (**C**), PL spectrum (**D**) and absorbance spectrum (**E**) of collagen, LC, MC, HC and QDs under 808-nm excitation.

Firstly, fluorescent characteristics of QDs-labeled collagen were measured and shown in Fig. 1. Bright-field images of collagen, LC, MC and HC are demonstrated in Fig. 1A. The color of collagen turned from transparent to dark brown after labeling by NIR-II QDs. NIR-II images of collagen and QDs-labeled collagen with the exposure time ranging from 0.5 to 4 ms were shown in Fig. 1B1–B5, which demonstrates bright NIR-II FL signals of QDs-labeled collagen, while unlabeled collagen was dark. The images of QDs were shown in Supplementary Fig. S1. The values of FL intensity were measured subsequently, which showed rising FL signals with the increase of exposure time. NIR-II FL signals reached the peak at 2 ms of exposure time and persisted with longer exposure times (Fig. 1C), verifying appropriate fluorescent characteristics of QDs-labeled collagen. Longer exposure times such as 15, 25, 50 and 100 ms led to overexposure (Supplementary Fig. S2). This result suggested that the labeling procedure did not affect the FL intensity of QDs. PL spectrum showed that QDs have an emission peak at around 1300 nm by the 808 nm excitation, which was consistent with our previous study [31] (Fig. 1D). The absorbance spectrum of collagen, QDs, LC, MC and HC all showed the peak of protein at around 280 nm [32] and LC, MC and HC had a similar absorbance spectrum (Fig. 1E). Notably, the emission peaks of QDs-labeled collagen had a blue shift in comparison with pure QDs (Fig. 1D), which could be ascribed to the increased interplanar spacing of QDs by labeling the crosslinked collagen, in agreement with our previous results [28, 33]. In summary, all degrees of crosslinked collagens were successfully labeled by NIR-II QDs, and high NIR-II signals of LC, MC and HC could be detected by *in vivo* NIR-II live imaging.

### The relationship between crosslinking degree and degradation rate of collagen based on *in vivo* NIR-II live imaging

**Figure 2.**
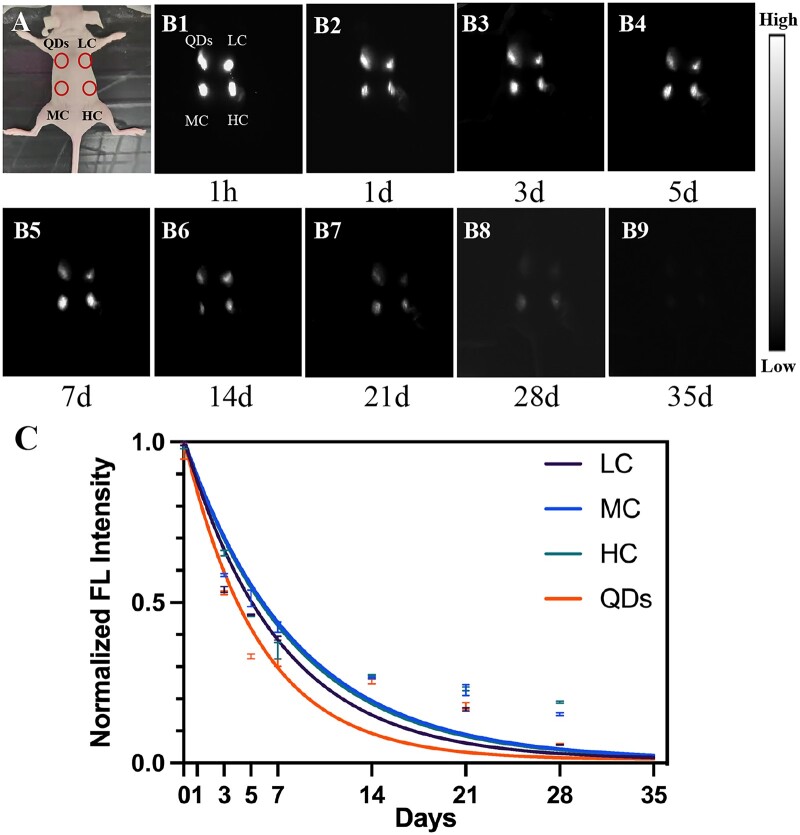
Collagen degradation in subcutaneous tissue. (**A**) Bright-field image of the subcutaneously injected mouse. (**B1-B9**) NIR-II fluorescence images were taken at 1 h, 1, 3, 5, 7, 14, 21, 28 and 35 days after injection of QDs, LC, MC and HC. (**C**) Corresponding FL intensity analysis (B) and colored solid curve representing the degradation rate of QDs, LC, MC and HC, respectively.

Firstly, the relationship between crosslinking degree and degradation rate of collagen was studied by *in vivo* NIR-II live imaging. As shown in Fig. 2A, QDs, LC, MC and HC were subcutaneously injected to four points at the back of the nude mouse. Then, NIR-II FL images were obtained from 1 h to 35 days post-injection (Fig. 2B). The bright spots were observed at injection points at 1 h post-injection (Fig. 2B1). The brightness of spots gradually decreased from 1 to 28 days (Fig. 2B2–B8) and no signals were detected in 35 days (Fig. 2B9). Normalized FL intensity was decreased over time (Fig. 2C); however, this attenuation was rapid in the first few days and flattened after 14 days. As shown in Fig. 2C, although MC had the highest FL intensity at the early stage of degradation (from 1 h to 7 days post-injection), HC turned out to have the highest FL intensity at the late stage of degradation (from 14 to 35 days post-injection). Meanwhile, there was no significant difference in the degradation rate of LC (*k* = 0.14), MC (*k* = 0.12) and HC (*k* = 0.12) based on the exponential decay model. Therefore, the change in crosslinking degree of collagen would not affect the degradation rate of collagen in subcutaneous tissue.

**Figure 3.**
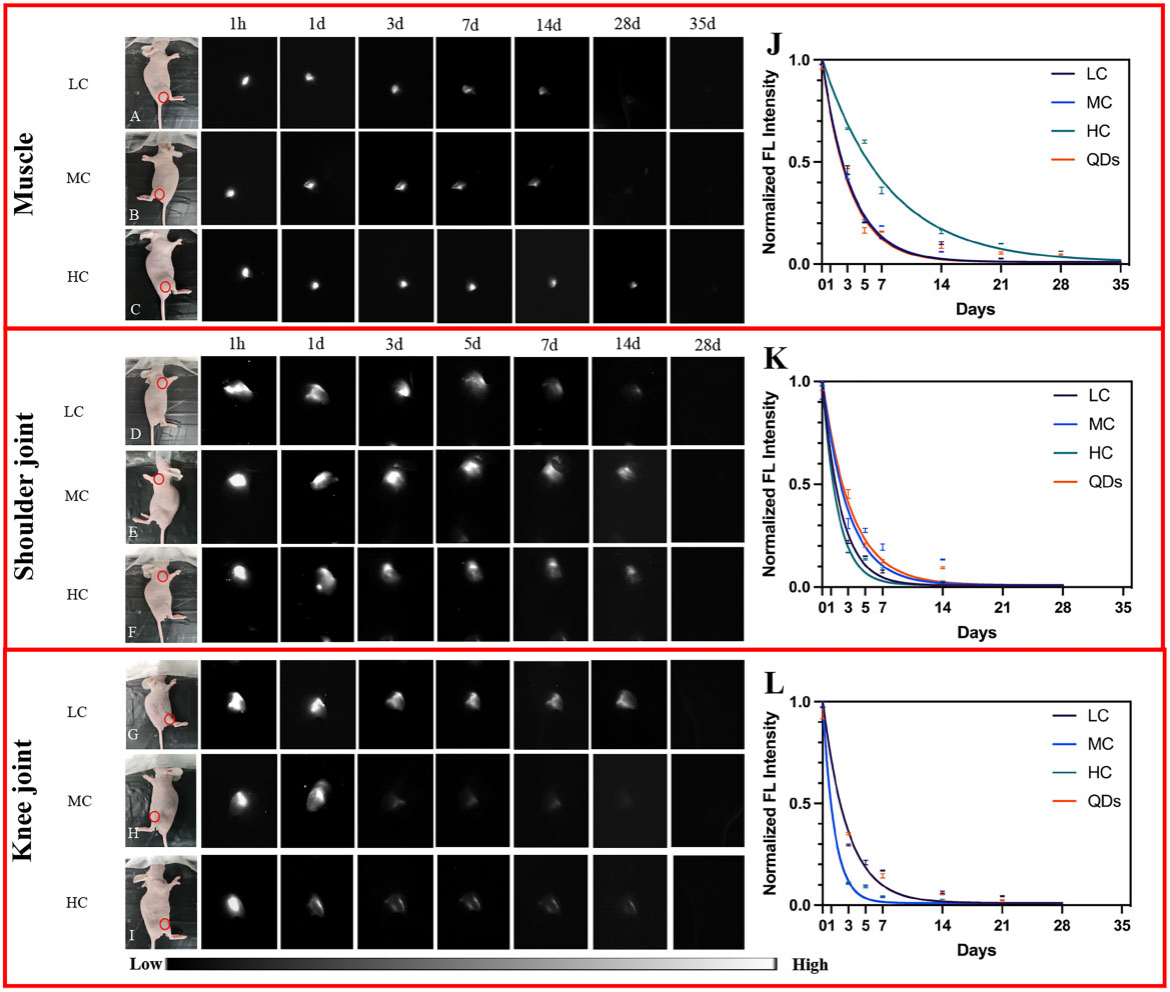
Collagen degradation in muscle, shoulder joint and knee joint. (**A–C**) Bright-field and NIR-II fluorescence images of LC (A), MC (B) and HC (C) degradation in muscle. (**D–F**) Bright-field and NIR-II fluorescence images of LC (D), MC (E) and HC (F) degradation in the shoulder joint. (**G–I**) Bright-field and NIR-II fluorescence images of LC (G), MC (H) and HC (I) degradation in the knee joint (circles: injection sites). (**J–L**) Corresponding normalized FL intensity analysis of collagen and degradation rate in muscle (J), shoulder joint (K) and knee joint (L).

Besides, QDs, LC, MC and HC were also injected into the muscle, shoulder and knee joints and the degradation activity was assessed. As shown in Fig. 3, the NIR-II signals of QDs, LC, MC and HC decreased gradually and no signals were detected at 35 days in muscle (Fig. 3A–C), while the signals disappeared at 28 days in both knee and shoulder joints (Fig. 3D–I). This result suggested that the degradation of collagen in joints was faster than that of subcutaneous tissue and muscle. Moreover, the signals of all three degrees of collagens decreased rapidly at the early stage and gradually flattened at the late stage (Fig. 3J–L), which suggested LC, MC and HC had a similar degradation pattern in the same body parts.

Noteworthy, although HC (*k* = 0.13) in muscle, MC (*k* = 0.34) in shoulder joint and LC (*k* = 0.35) in knee joint had a slower degradation rate, respectively, the others had similar degradation rates despite the crosslinking degree in the corresponding body parts (Fig. 3J–L). Thus, the degradation rate of collagen did not change according to the crosslinking degree, when injected to the same body parts.

### The relationship between body parts and degradation rate of collagen based on *in vivo* NIR-II live imaging

**Figure 4.**
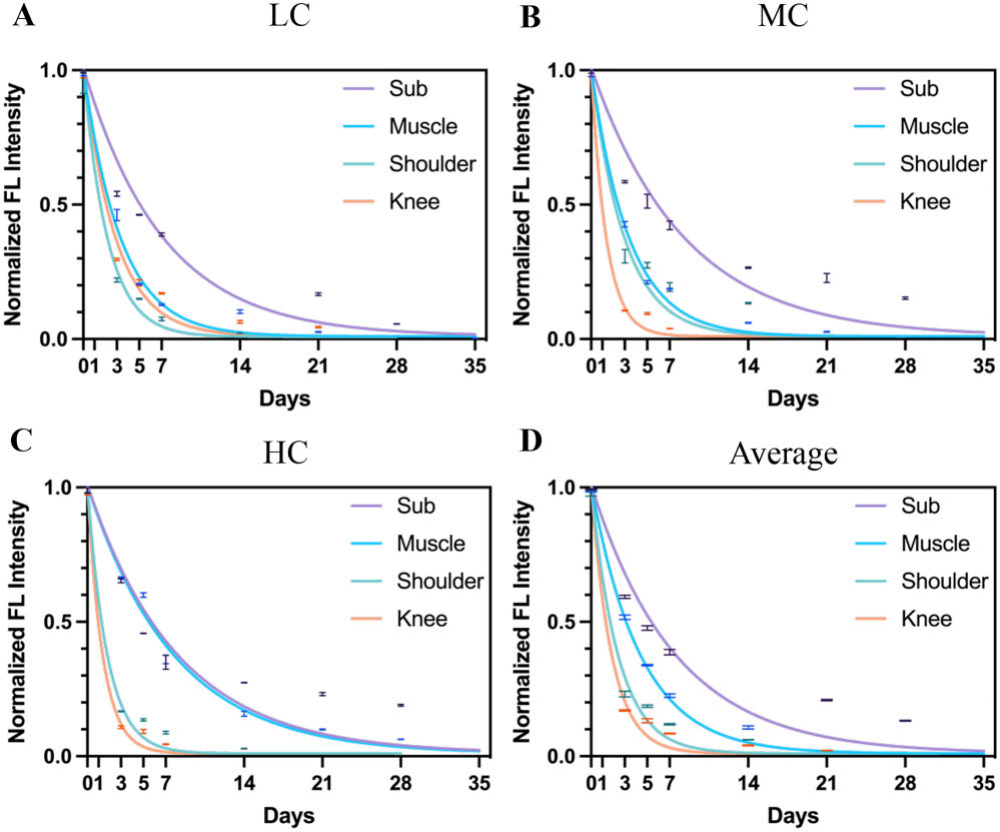
LC, MC and HC degradation in different tissue. Normalized FL intensity and degradation rate of LC (**A**), MC (**B**) and HC (**C**) in subcutaneous tissue, muscle, shoulder joint and knee joint ranging from 1 h to 35 days. (**D**) Average normalized FL intensity and degradation rate of collagen in subcutaneous tissue, muscle, shoulder joint and knee joint, respectively.

The previous result revealed that the total degradation time of collagen in joints was faster than that of subcutaneous tissue and muscle. Therefore, the specific degradation rates in four tested positions were further analyzed to investigate the relationship between the implanted body parts and collagen degradation. As shown in Fig. 4A–C, all kinds of collagen had substantially flatter curves in subcutaneous tissue compared to joints, while the HC in subcutaneous (*k* = 0.13) and muscle (*k* = 0.12) had similar degradation rates (Fig. 4C). Specifically, the subcutaneous tissue had the slowest degradation rates with all tested collagen, and the degradation rates of LC, MC and HC were 0.14, 0.12 and 0.12, respectively. Moreover, the degradation rates of HC in the shoulder joint and MC and HC in the knee joints were the fastest, which were 0.56, 0.74 and 0.73. Therefore, it was suggested that collagen degraded at the slowest rate in the subcutaneous tissue and the fastest rate in the knee joint. Likewise, the average degradation rate of collagen also confirmed that subcutaneous tissue (*k* = 0.13) and muscle (*k* = 0.23) had a slower degradation rate compared to shoulder (*k* = 0.42) and knee (*k* = 0.55) joints, as shown in Fig. 4D. In summary, it was suggested that the microenvironment of implantation markedly affected the degradation pattern of collagen.

### *Ex vivo* imaging of NIR-II QDs-labeled collagen

**Figure 5.**
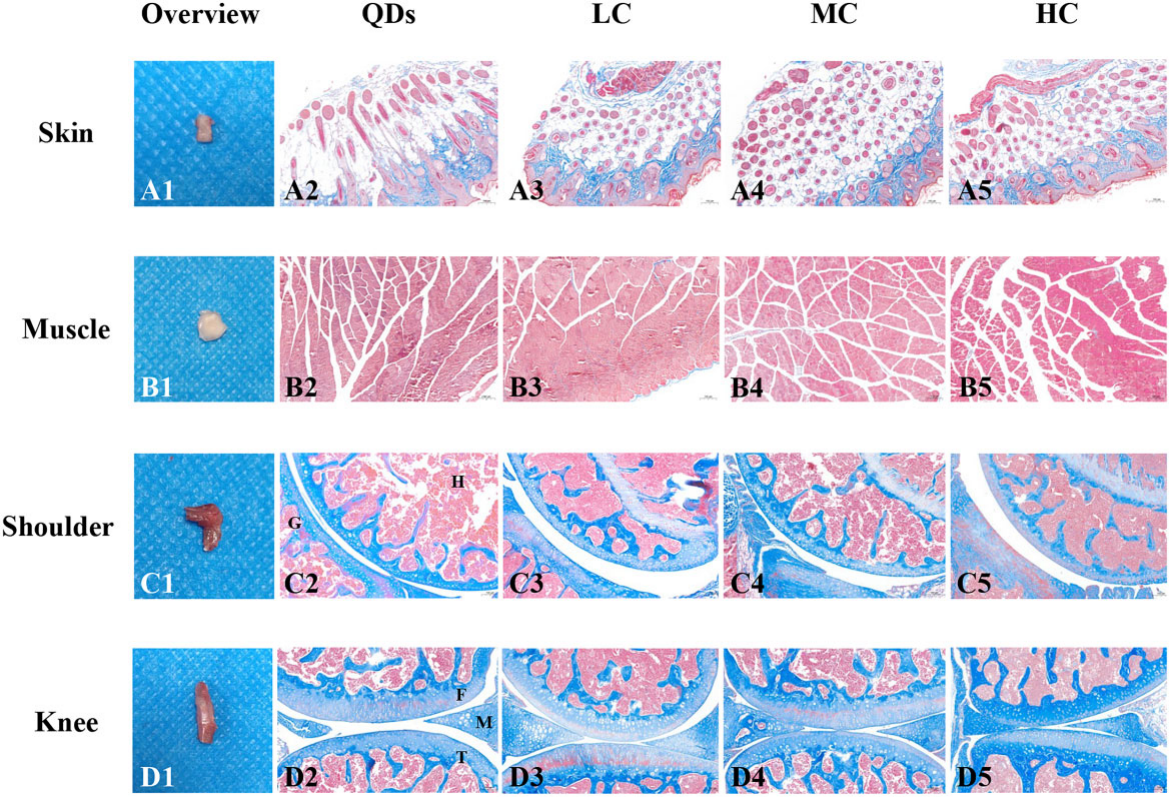
Macroscopic and microscopic images of collected tissues after the signals disappeared. (**A1–D1**) Macroscopic images of collected skin (35 days), muscle (28 days for LC, MC; 35 days for HC), shoulder joint (28 days) and knee joint (28 days). (**A2–D2**) Micrographs of Masson’s trichrome-staining slices of tissues injected with (**A2–D2**) QDs, (**A3–D3**) LC, (**A4–D4**) MC and (**A5–D5**) HC. H, humerus; G, glenoid; F, femur; T, tibia; M, meniscus. Magnification: ×100 for (**A2–A5**), (**B2–B5**), (**C2–C5**), (**D2–D5**).

Skin, muscle, shoulder and knee joints were collected from each group to investigate whether the collagen degraded completely as well as the effect of the current method on injected sites. Figure 5A1–D1 showed the macroscopic images of tissues. No redness or edema was found in collected skin and muscle, suggesting there was no obvious inflammation in these tissues (Fig. 5A1 and B1). Shoulder joints and knee joints had normal joint morphology without malformation (Fig. 5C1 and D1). Masson’s trichrome-staining images of tissues injected with LC (Fig. 5A3–D3), MC (Fig. 5A4–D4) and HC (Fig. 5A5–D5) were compared to those that were injected with QDs (Fig. 5A2–D2). Ordered blue-stained collagen was observed in LC, MC and HC groups (Fig. 5A3–A5), which was similar to QDs group (Fig. 5A2), suggesting inherent collagen of host tissue. Red-stained myofiber was found in Fig. 5B3–B5 without blue-stained collagen, suggesting they degraded completely in muscle. Figure 5C3–C5 and D3–D5 showed there were no blue-stained LC, MC or HC in the shoulder joint capsules and knee joint capsules. The morphology of tissue was similar to that of the QDs-injected group, with no exudation or inflammatory cell infiltration. In summary, LC, MC and HC degraded completely in subcutaneous tissue, muscle, shoulder and knee joints without obvious inflammation.

**Figure 6.**
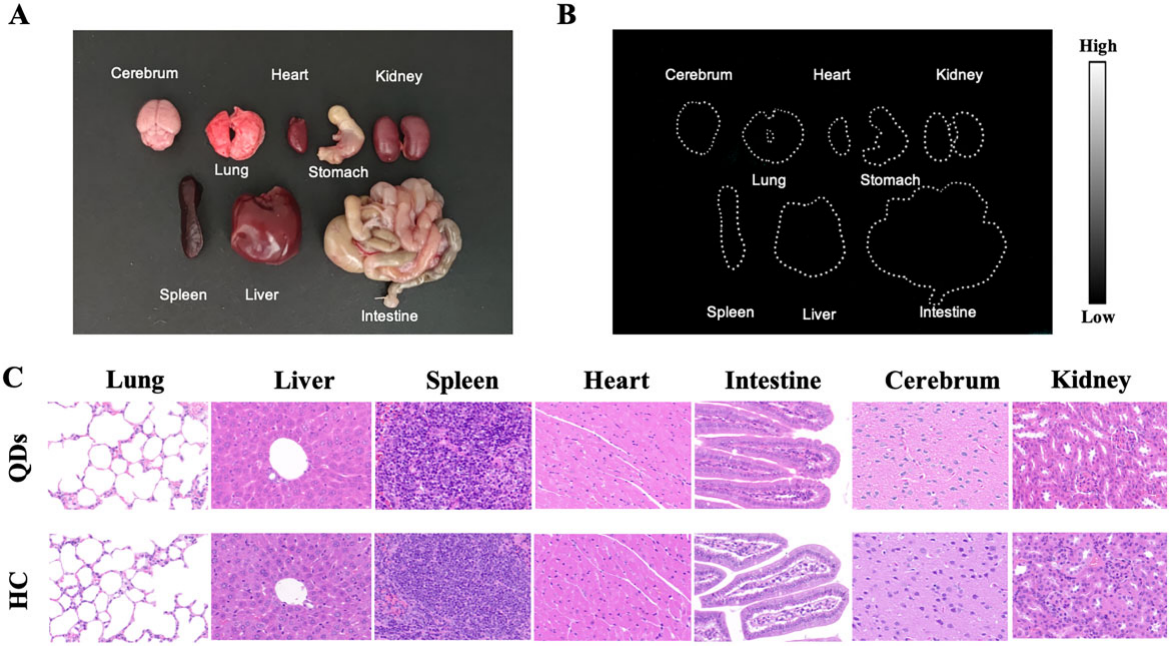
Biosafety of QDs-labeled collagen. (**A**) Bright-field image of major organs including cerebrum, lung, heart, stomach, kidney, spleen, liver and intestine from mouse injected with QDs-labeled collagen. (**B**) NIR-II fluorescence image of major organs. (**C**) HE staining of organs from QDs-injected mice and HC-injected mice. Magnification: ×400.

Major organs including the cerebrum, lung, heart, stomach, kidney, spleen, liver and intestine were harvested after no NIR-II FL signals were detected. The bright-field image was shown in Fig. 6A, which demonstrated no significant morphological changes. Then NIR-II FL images of organs were obtained with no obvious signals found in major organs (Fig. 6B). This result indicated that QDs-labeled collagen was metabolized well and did not aggregate in major organs after 35 days of injection. Moreover, histological analysis was performed to investigate the influence of QDs-labeled collagen on the microstructure of these organs, since the previous study confirmed the low biotoxicity of pure QDs [34]. HE staining of organs harvested from QDs-injected BALB/C nude mice and that from HC-injected mice were shown in Fig. 6C. No obvious signs of morphological changes and inflammation were found in the organs of HC-injected mice compared to QDs-injected mice. It could be concluded that QDs-labeled collagen did not deposit to or harm the major organs.

**Scheme 2.**
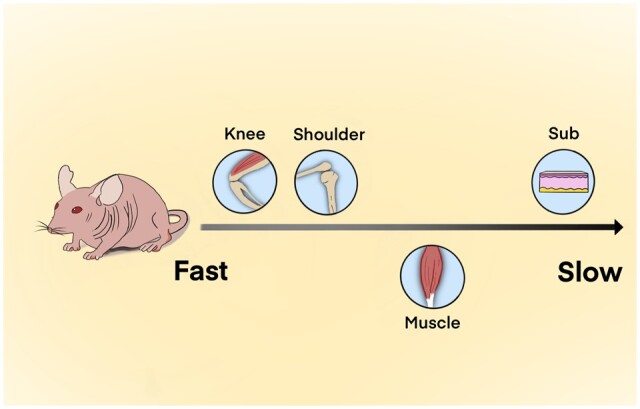
Comparison of collagen degradation rate in different body parts of the mouse model.

## Discussion

In recent decades, NIR-II live imaging has emerged as a perfect choice for *in vivo* live imaging because of its high tissue penetration and low light-scattering characteristics [34–42]. In fact, NIR-II imaging has successfully monitored the degradation of many biomedical implants [22, 43] with high resolution. This study also successfully monitored the collagen degradation patterns by NIR-II *in vivo* live imaging, confirming its potential for a broader range of biomedical products due to its supreme imaging ability. Therefore, it could be a potential standard *in vivo* monitoring modality for implanted biomedical products.

It has been previously shown that a higher crosslinking degree of collagen would lead to a slower degradation rate in *ex vivo* conditions due to the decreased enzyme-reacting points consumed by the crosslinking [16, 17, 44]. However, this *in vivo* study showed that the crosslinking degree of collagen did not affect the degradation rate in most conditions. This indicates that some biological factors, such as varying degrees of collagenase, might be neglected in the *ex vivo* study, while they play a critical role in collagen degradation. Interestingly, it has been reported that higher crosslinking degree of collagen would stimulate the dermal fibroblasts to secrete higher level of collagenase [19], and crosslinking would improve the macrophage attachment [44]. A possible explanation is that the collagen would induce an acute reaction which could make the cells to accumulate to produce more collagenase. Although highly crosslinked collagen had a small amount of enzyme reaction points, the high level of collagenase hindered it to remain longer. In contrast, although the lower crosslinking degree of collagen had less amount of enzyme reacting points, it also made the cells secrete a lower level of collagenase. As a result, no significant differences were found between the degradation rates of the three crosslinking degrees of collagens. Therefore, the balance between the enzyme reacting points and the level of collagenase produced by cells would be critical for collagen degradation.

This study showed that three kinds of collagens degraded most slowly in the subcutaneous tissue and most rapidly in the joints regardless of the crosslinking degree, suggesting that the microenvironment of body parts might play a critical role in this process (Scheme 2). As mentioned above, collagenase is a key factor that affects collagen degradation and it might explain the varying degradation rates in different body parts. The synovial fluid in the joint capsule is rich in macrophages [45], the activity of which is correlated with the collagenase activity [44]. In addition, the spaces in the synovial cavity of joints could support more sufficient interactions between collagenase and collagen [44, 46] compared to subcutaneous tissue and muscle. Large amounts of macrophages and a relatively wide interaction space might lead to a quick metabolism of collagen in joints than in subcutaneous tissue and muscle despite the crosslinking degree. Therefore, it could be concluded that the collagen degradation *in vivo* might be mainly decided by the level of collagenase, and it is the microenvironment of the body parts, which is responsible for the level of collagenase.

## Conclusion

In this study, both the crosslinking degree and the implantation sites of collagen have been simultaneously taken into consideration, to monitor and evaluate the *in vivo* degradation pattern of collagen using NIR-II live imaging. Firstly, three kinds of crosslinking degrees of collagens, including HC, MC and LC, were successfully labeled by NIR-II fluorescent QDs and then were injected into the subcutaneous tissue, muscle, shoulder and knee joints of the mouse model. The NIR-II imaging showed that the implanted collagen had a slower and longer degradation in the subcutaneous tissue and muscle in comparison with that in the joints. Interestingly, the degradation rate of collagen in subcutaneous tissue, muscle, shoulder and knee joints did not depend on the crosslinking degree of collagen. In summary, this study indicated that the degradation pattern of collagen was closely related to the body parts rather than the chemical properties of collagen in the mouse model, implying that the biological microenvironment of the implanted sites should be considered first when designing collagen-based products for biomedical applications.

## Supplementary Material

rbac102_Supplementary_Data

## Data Availability

The data that support the findings of this study are available from the corresponding author upon reasonable request.
