# Supplementary material for: NIR-II live imaging study on the degradation pattern of collagen in the mouse model
Source: Regen Biomater. 2022 Dec 13;10:rbac102. doi: 10.1093/rb/rbac102 (PMC9847529; doi:10.1093/rb/rbac102)
Supplement: rbac102_Supplementary_Data [file rbac102_supplementary_data.docx]

**Support information**

**NIR-II live imaging study on the degradation pattern of collagen in the mouse model**

Huizhu Li^1#^, Xinxian Meng^2#^, Huaixuan Sheng^1#^, Sijia Feng^1^, Yuzhou Chen^1^, Dandan Sheng^1^, Liman Sai^3^, Yueming Wang^4^, Yan Wo^4^, Shaoqing Feng^2^, Hossein Baharvand^5,6^, Yanglai Gao^7*^, Yunxia Li^1*^, Jun Chen^1*^

^1^ Sports Medicine Institute of Fudan University, Department of Sports Medicine, Huashan Hospital, Fudan University, Shanghai 200040, China.

^2^ Department of Plastic and Reconstructive Surgery, Shanghai Ninth People’s Hospital, School of Medicine, Shanghai Jiao Tong University, 639 Zhizaoju Rd, Shanghai 200011, P.R. China

^3^ Department of Physics, Shanghai Normal University, Shanghai 200234, China.

^4^ Department of Anatomy and Physiology, School of Medicine, Shanghai Jiao Tong University, Shanghai 200025, China

^5^ Department of Stem Cells and Developmental Biology, Cell Science Research Center, Royan Institute for Stem Cell Biology and Technology, ACECR, Tehran, Iran;

^6^ Department of Developmental Biology, School of Basic Sciences and Advanced Technologies in Biology, University of Science and Culture, Tehran, Iran.

^7^ Hexi College, Zhangye 73400, Gansu, P.R. China

*Correspondence address: Email: 1811292675@qq.com (Y.G.); liyunxia912@aliyun.com (Y. L.) ; biochenjun@fudan.edu.cn (J. C.)

^#^These authors contributed equally to this work.


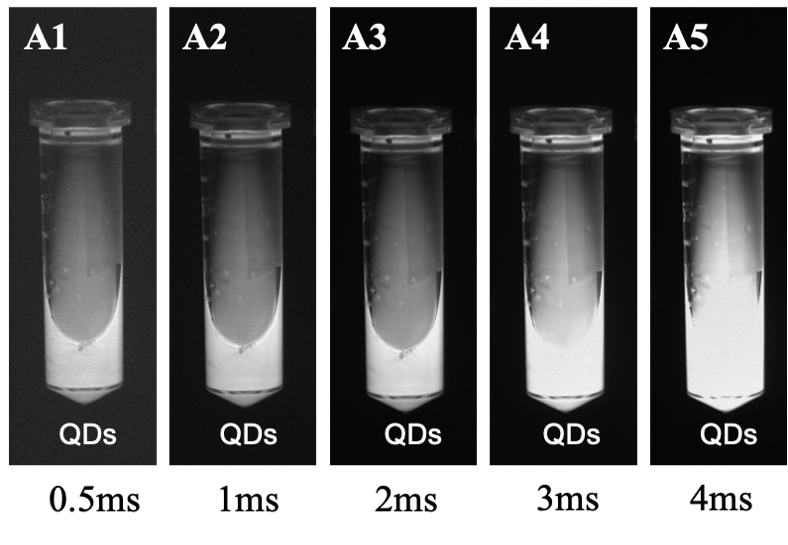


Figure S1. NIR-II images of QDs at the exposure time from 0.5ms to 4ms.


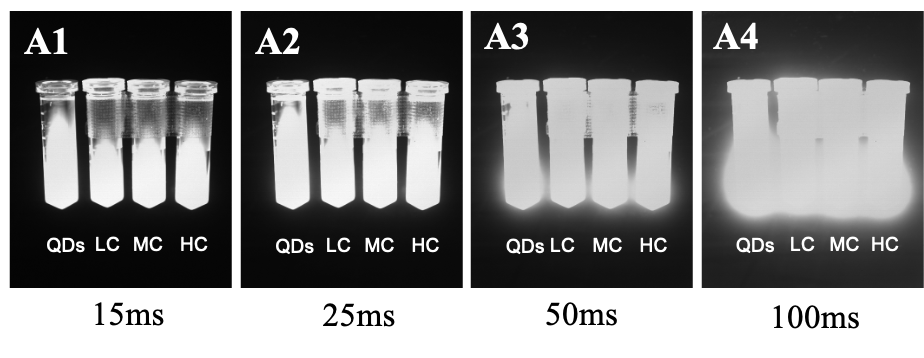


Figure S2. NIR-II images of QDs, LC, MC, HC at the exposure time of 15ms, 25ms, 50ms and 100ms.
